# On the Diverse Functions of Electrical Synapses

**DOI:** 10.3389/fncel.2022.910015

**Published:** 2022-06-09

**Authors:** Mitchell J. Vaughn, Julie S. Haas

**Affiliations:** Department of Biological Sciences, Lehigh University, Bethlehem, PA, United States

**Keywords:** electrical synapse, gap junction, connexin, synchrony, integration

## Abstract

Electrical synapses are the neurophysiological product of gap junctional pores between neurons that allow bidirectional flow of current between neurons. They are expressed throughout the mammalian nervous system, including cortex, hippocampus, thalamus, retina, cerebellum, and inferior olive. Classically, the function of electrical synapses has been associated with synchrony, logically following that continuous conductance provided by gap junctions facilitates the reduction of voltage differences between coupled neurons. Indeed, electrical synapses promote synchrony at many anatomical and frequency ranges across the brain. However, a growing body of literature shows there is greater complexity to the computational function of electrical synapses. The paired membranes that embed electrical synapses act as low-pass filters, and as such, electrical synapses can preferentially transfer spike after hyperpolarizations, effectively providing spike-dependent inhibition. Other functions include driving asynchronous firing, improving signal to noise ratio, aiding in discrimination of dissimilar inputs, or dampening signals by shunting current. The diverse ways by which electrical synapses contribute to neuronal integration merits furthers study. Here we review how functions of electrical synapses vary across circuits and brain regions and depend critically on the context of the neurons and brain circuits involved. Computational modeling of electrical synapses embedded in multi-cellular models and experiments utilizing optical control and measurement of cellular activity will be essential in determining the specific roles performed by electrical synapses in varying contexts.

## Introduction

Across the nervous system, neurons couple to other neurons at gap junctions formed by plaques of paired and docked hemichannel pores composed of connexin or innexin proteins (Phelan et al., 1996, 1998; Starich et al., 1996; Condorelli et al., 1998; Landesman et al., 1999; Rash et al., 2000, 2001a,b; Stebbings et al., 2000; Söhl and Willecke, 2003) that allow ions to pass between neurons. Gap junctions are the biophysical substrate for the neurophysiological component of electrical synapses (Furshpan and Potter, 1957, 1959; Watanabe, 1958; Bennett, 1966), which couple neurons in the mature mammalian brain. These unique structures enable current to flow directly between neurons without relying on energetically costly neurotransmitters or a presynaptic spike in order to initiate inter-neuronal communication. Similar to their more abundant counterpart, chemical synapses, the function of electrical synapses is of great interest in determining how neurons integrate inputs and information.

Much early work focused on the potential for electrical coupling to synchronize firing between neurons. Electrical synapses pass ions proportionally to the difference in membrane voltage between coupled neurons, and their most basic effect is to reduce that difference, resulting in minimization of differences in voltage or activity. This is thought to be the simplest, though not sole, mechanism that underlies synchrony between coupled neurons. Synchrony, generally considered, can be appreciated at the level of ongoing repetitive activity, or for individual spikes. Synchrony of spiking activity in broader heterogenous networks, while more complex in mechanisms, often relies on contributions from the inhibitory neurons that gap junctions frequently couple. Thus, electrical synapses contribute to synchrony both directly and indirectly. Because neuronal electrical synapses take diverse values of strength amongst brain areas, their effects only rarely approach the perfect synchronization that would occur between coupled neurons with infinitely strong electrical synapses. This opens the door to a variety of diverse effects mediated by electrical synapses within neural circuitry. Previous reviews have covered aspects of electrical synapses (Bennett and Zukin, 2004; Connors and Long, 2004; Haas et al., 2016; Connors, 2017; Nagy et al., 2018; Alcami and Pereda, 2019; Trenholm and Awatramani, 2019; Curti et al., 2022). Here, we detail the progress in understanding the functions of electrical synapses within, and as a result of, coupled circuits and networks. We organize our review by the different functions of electrical synapses, followed by comments on future directions for studying electrical synapses. Because so much work involves interrogating the role of electrical synapses in synchrony of rhythmic activity, that section is divided into subsections based on the source of the evidence.

### Synchrony of Rhythmic Activity

**FIGURE 1:**
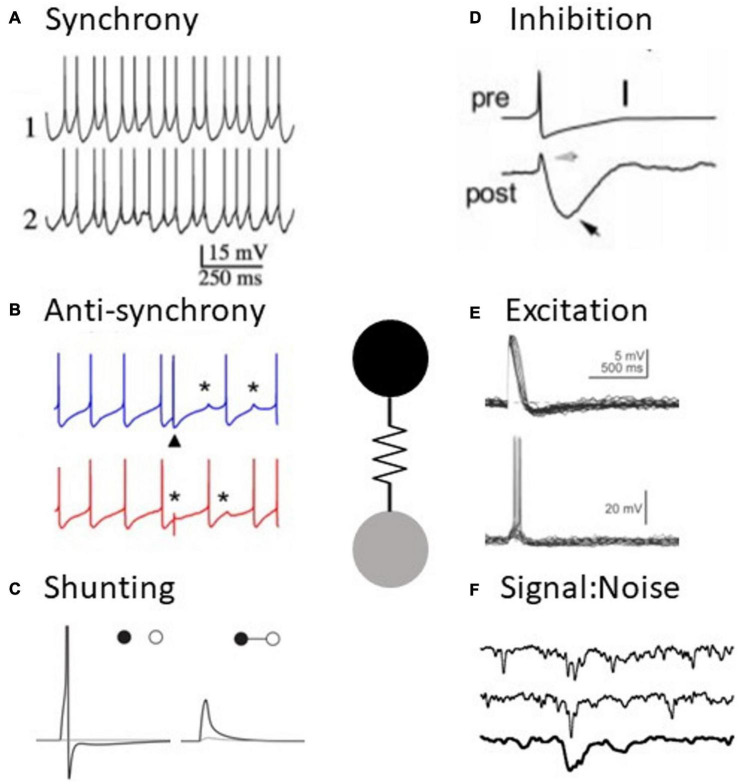
Electrical synapses have diverse functions in neural processing. **(A)** Example of synchronous activity in coupled neurons (adapted from Long et al., 2004). **(B)** Example of anti-synchronous firing in coupled neurons (adapted from Vervaeke et al., 2010). **(C)** An electrical synapse (right) shunts an excitatory signal to one cell to subthreshold levels, compared to the uncoupled case (left) (adapted from Hjorth et al., 2009). **(D)** Slow spike afterhyperpolarizations appear as inhibition in a coupled neuron (adapted from Galarreta and Hestrin, 2001). **(E)** Spikes result in excitation in a coupled neuron (adapted from Apostolides and Trussell, 2014). **(F)** Example of a signal amidst noise in retinal neurons, impacted by the presence of electrical synapses (adapted from Dunn et al., 2006).

Reports of synchrony are a hallmark of electrical synapse work across neural tissue (Figure 1A). Watanabe (1958) made the earliest inference, noting that the synchronous subthreshold fluctuations of lobster heart ganglion cells were a function of common presynaptic inputs and electrical coupling. This was followed by pioneering demonstrations of electrical coupling by Bennett (1966), who inferred that electrical synapses were likely to be associated with synchronization of activity, based on observations of electrical coupling in fish electromotor neurons and toad swim bladder motor neurons. Later, an early report of synchrony in feline inferior olive was associated with the presence of electrical synapses (Llinas et al., 1974). This was eventually followed by paired-patch recordings that identified electrical coupling between inhibitory interneurons in murine cortex and also found that coupled neurons were inclined to spike together when depolarized (Galarreta and Hestrin, 1999; Gibson et al., 1999; Beierlein et al., 2000). Electrical synapses have long been known to contribute to synchrony in crustacean neural networks (Eisen and Marder, 1982; Hooper and Marder, 1987; Gutierrez et al., 2013).

#### Neocortex

Cortical rhythms are diverse and include gamma-range oscillations, which are linked to higher-order function (Benchenane et al., 2011); beta oscillations, which are involved in sensorimotor processes (Kilavik et al., 2013); theta oscillations associated with learning, memory, spatial navigation, and speech (Benchenane et al., 2011; Giraud and Poeppel, 2012); delta oscillations that are related to speech processing and decision making (Giraud and Poeppel, 2012; Nácher et al., 2013); and alpha oscillations that are tied to attention (Buzsáki and Draguhn, 2004; Hanslmayr et al., 2011). In electrically coupled cortical interneurons, simultaneous depolarization drives coordinated spiking (Gibson et al., 1999, 2005; Beierlein et al., 2000; Blatow et al., 2003; Mancilla et al., 2007; Hu and Agmon, 2015). Carbachol-induced spiking is synchronous in coupled inhibitory neurons as well, and that synchrony is correlated with electrical synapse conductance, while uncoupled neurons displayed no correlation in spiking (Caputi et al., 2009). Similarly, correlated spiking induced by ACPD or carbachol is desynchronized by connexin36 knockout or pharmacological blockade of gap junctions (Deans et al., 2001; Blatow et al., 2003). For layers 2 and 3 basket cells, gamma frequency stimulation in one cell entrains gamma frequency spiking in coupled basket cells, albeit with a phase lag of about 10 ms; electrical coupling in addition to GABAergic connections shortened gap junction mediated postsynaptic potentials and promoted synchronous gamma activity (Tamás et al., 2000). Synchronization of cortical interneurons can entrain synchrony of rhythmic spiking in cortical pyramidal neurons (Whittington et al., 1995, 2000; Traub et al., 1996; Whittington and Traub, 2003; Cardin, 2016); synchronized network rhythms may be important for sensory processing, working memory, and attention (Buzsáki and Draguhn, 2004; Wang, 2010; Benchenane et al., 2011; Hanslmayr et al., 2011).

#### Thalamus

The thalamic reticular nucleus (TRN) receives excitatory input from ascending thalamic relay nuclei (Jones, 1975; Ohara and Lieberman, 1985; Fosse et al., 1986; Fitzpatrick et al., 1994; Liu and Jones, 1999), feedback excitation from layers 5 and 6 of cortex (Jones, 1975; Bromberg et al., 1981; Fonnum et al., 1981; Feig and Harting, 1998; Zhang and Jones, 2004), and a myriad of modulatory input (Wilson, 1985; Cornwall et al., 1990; Gandia et al., 1993; Reardon and Mitrofanis, 2000; Freeman et al., 2001; Prensa and Parent, 2001; Anaya-Martinez et al., 2006; Zikopoulos and Barbas, 2012; Leon-Dominguez et al., 2013); the exclusively GABAergic neurons of the TRN provide the main source of inhibitory drive onto thalamic nuclei (Scheibel and Scheibel, 1966; Jones, 1975; Houser et al., 1980), and thus control thalamocortical relay of sensory information. The TRN is integral in thalamocortical spindle oscillations during sleep and memory formation during sleep (Pinault, 2004; Steriade, 2005; Halassa et al., 2011; Latchoumane et al., 2017). Paired depolarization of coupled TRN neurons drives correlated spiking (Landisman et al., 2002; Haas and Landisman, 2011), and depolarization resulting from ACPD application also causes correlated spiking (Long et al., 2004). The cross-correlation of mGluR-induced subthreshold rhythms is positively correlated with the conductance of the electrical synapse within the pair.

#### Hippocampus

The nested gamma and theta rhythms of the hippocampal formation are essential to its spatial and memory functions (Colgin and Moser, 2010), and hippocampal GABAergic neurons are connected *via* dendrodendritic gap junctions (Fukuda and Kosaka, 2000). Hippocampus basket cells initiate gamma oscillations when depolarized with potassium, glutamate, kainite, or carbachol; the measured power of those gamma oscillations through field potentials are reduced with gap junction blockers or connexin knockout (Hormuzdi et al., 2001; Traub et al., 2001). Furthermore, IPSCs onto pyramidal cells become more variable in connexin knock outs (Hormuzdi et al., 2001). The effect of gap junctions synchronizing gamma oscillations was reinforced by a following study recording field potentials of CA1 pyramidal neurons *in vivo*, where connexin36 knockout reduced the power of gamma oscillations (Buhl et al., 2003).

Synchronous high frequency (<150 hz) bursts of activity occur in hippocampal pyramidal neurons, and gap junctions are necessary for the high frequency bursts to occur *in vitro* (Draguhn et al., 1998), although this was not confirmed *in vivo* (Buhl et al., 2003). Seizing activity in hippocampal slices induced by Ca^2+^ free ACSF is reduced and desynchronized by weakening electrical coupling through acidification (Perez-Velazquez et al., 1994). Similarly, another hippocampal seizure model with no Mg^2+^ and 4-aminopyridine in the ACSF had its population bursts greatly reduced by gap junction blockers, suggesting that electrical synapses were partially responsible for simultaneous activity seen in the seizure model (Ross et al., 2000).

#### Cerebellum

The cerebellum is responsible for timing of motor control, and precise temporal precision of spiking is key to that function. Both low frequency (7–30 hz) oscillations and high frequency (>40 hz) oscillations have been observed in the cerebellum and may contribute to motor execution and learning (D’Angelo et al., 2009). *In vitro*, spontaneous spiking in coupled Golgi inhibitory interneurons of the cerebellum is correlated. Further, coupled Golgi neurons oscillate synchronously or spike synchronously when depolarized with kainate (Dugué et al., 2009). Computational modeling of Golgi neurons suggests that transfer of the after hyperpolarization through the gap junction is critical for synchronized spiking. The afterhyperpolarization (AHP) is inhibitory to the coupled cell and determines the delay period after which a coupled neuron can next spike (Dugué et al., 2009). *In vivo* cell-attached recordings demonstrated that coupled Golgi interneuron spiking is correlated within a few ms (van Welie et al., 2016); knock-out of connexin36 results in uncorrelated spiking. Paired recordings showed that spiking in one neuron resulted in a depolarizing spikelet in the other, and in some instances, the spikelet was sufficient to drive a correlated spike within milliseconds. Altogether, these heroic *in vivo* experiments suggest that coupled Golgi interneurons spike together due to electrical synapses correlating subthreshold potentials and by driving a coupled neighbor past threshold through an excitatory spikelet.

#### Inferior Olive

The coupled neurons of the inferior olive provide error-signal input to cerebellar Purkinje cell dendrites and exhibit strong subthreshold and spiking oscillations in the 1–10 Hz range of frequency (Armstrong et al., 1968; De Zeeuw et al., 1998). Paired recordings of inferior olive (IO) neurons show that spontaneous spiking and subthreshold oscillations are highly correlated amongst coupled neurons. Knockout of connexin36 causes an increase in uncorrelated spiking and desynchronization of subthreshold oscillations, which still persist (Long et al., 2002; Leznik and Llinas, 2005). Long et al. (2002) suggest that coupled IO neurons spike synchronously in part due to coupled neurons faithfully spiking at the peak of membrane fluctuations, where decoupled neurons sometimes spike in the trough of their oscillation. Electrical synapses reduce excitability by shunting current, and therefore, they make IO neurons less likely to spike during the trough of a subthreshold membrane oscillation.

Synchronization of IO neurons *via* gap junctions affects the downstream synchrony of cerebellar neurons. *In vivo* injections of gap junction blockers into the IO or knockout of connexin36 reduces synchrony of complex spikes in postsynaptic Purkinje cells (Blenkinsop and Lang, 2006; Marshall et al., 2007) and interferes with motor learning (Van Der Giessen et al., 2008). This effect is presumably the result of desynchronized activity in the uncoupled IO.

#### Suprachiasmatic Nucleus

The suprachiasmatic nucleus (SCN) is a central regulator of circadian rhythms. Dye-coupling, which images the spread of gap junction-permeable dyes through coupled networks, is greatest during the sleep cycle, when there is more synchronous activity, than during the active cycle (Colwell, 2000). Coupling is positively correlated with synchronous spiking, while spiking was uncorrelated in uncoupled neurons (Long et al., 2005). The circadian rhythms of connexin36 knockout mice are irregular, so the synchronous firing during the wake cycle may be a component of circadian rhythm maintenance (Long et al., 2005). Long et al. (2005) also showed evidence that coupling between cells changes in strength over 24 h and that knockout rats lose rhythmicity of circadian activity in 24 h dark conditions.

#### Brainstem

Evidence for the synchronizing effects of gap junctions in brainstem is mixed. One study using adult rats found that gap junction blockers decreased synchrony of phrenic bursts (Solomon et al., 2003). In contrast, an earlier study found that gap junction blockers in neonatal brain stem slices showed that gap junctions reduce synchronized activity of the phrenic nerve in the short term (Bou-Flores and Berger, 2001). In the mesencephalic trigeminal nucleus, strong electrical synapses can drive a quiescent neighbor to spike within 2 ms of the spikes of coupled neurons. Further, subthreshold depolarizations of coupled neurons resulted in membrane potential oscillations that were correlated between coupled pairs (Curti et al., 2012). Synchronization of the mesencephalic trigeminal nucleus could be important for coordinated inputs to relax the jaw.

#### Spinal Cord

Electrical coupling is present between juvenile motor neurons in rats, but it does not persist with maturation (Walton and Navarrete, 1991; Chang et al., 1999). Juvenile motor neuron spiking is correlated, even the presence of TTX, but spiking becomes uncorrelated with pharmacological blockade of gap junctions (Tresch and Kiehn, 2000; Personius et al., 2001). Electrical synapses may have an important role in guiding development of motor neurons by promoting synchronous activity. Inhibition of acetylcholine release by motor neurons during development causes electrical synapses to persist into adolescence, suggesting that activity at the neuromuscular junction communicates the need to remove electrical synapses (Pastor et al., 2003).

#### Olfactory Bulb

Mitral cells in the olfactory bulb synchronously oscillate at 30–80 hz when stimulated with one or more odorants (Kashiwadani et al., 1999). Oscillations in the olfactory bulb have a role in odorant discrimination, and it is speculated that synchronous activity facilities the summation of EPSPs in piriform cortex (Schoppa and Urban, 2003; Rojas-Líbano and Kay, 2008). Electrical synapses contribute to synchrony in the olfactory bulb; depolarizing coupled mitral cells in the olfactory bulb drives correlated spiking that is absent when connexin36 is knocked out (Christie et al., 2005).

#### Retina

Retina is a major hub where electrical synapses exert influence on processing light signals. All layers of the retina express gap junctions, and their electrical synapse strength is regulated by brightness *via* dopamine, nitric oxide, and adenosine (Hampson et al., 1992; McMahon and Brown, 1994; Mills and Massey, 1995; Lee et al., 2002; Mills et al., 2007; Ribelayga et al., 2008; Bloomfield and Volgyi, 2009; Kothmann et al., 2009; Li et al., 2013; Jacoby et al., 2018; Trenholm and Awatramani, 2019). Electrical synapse regulation in retina is critical for adaptation to different light intensities.

The developing retina undergoes slow waves of activity that spread across ganglion cells with an interval on the order of minutes (Firth et al., 2005). Retinal waves require electrical synapses and L-type Ca^2+^ channels. It has been inferred that electrical synapses propagate the wave by transferring excitation driven by L-type Ca^2+^ channels (Singer et al., 2001; Firth et al., 2005). Further, electrical synapses contribute to the responsiveness of developing retina to light, and those electrical synapses are depressed by retinal waves and dopamine release (Arroyo and Feller, 2016).

*Computational modeling* of electrical synapses robustly supports the role of electrical synapses in synchronization (Mulloney et al., 1981; Sherman and Rinzel, 1992; Traub, 1995; Chow and Kopell, 2000; Moortgat et al., 2000; Velazquez and Carlen, 2000; Nomura et al., 2003; Pfeuty et al., 2003; Bem and Rinzel, 2004; Kopell and Ermentrout, 2004; Mancilla et al., 2007; Ostojic et al., 2009). Modeling suggests that electrical synapses can switch from promoting synchrony to anti-synchrony in different contexts (Mulloney et al., 1981; Sherman and Rinzel, 1992; Chow and Kopell, 2000; Lewis and Rinzel, 2003; Nomura et al., 2003; Pfeuty et al., 2003; Bem and Rinzel, 2004; Gibson et al., 2005; Mancilla et al., 2007). Some models point to voltage equalization as a key part of electrical synapse promotion of synchrony (Sherman and Rinzel, 1992; Montbrió and Pazó, 2020). In addition, models have shown that synchrony is promoted by strong electrical synapses, while asynchrony is promoted by weak electrical synapses (Chow and Kopell, 2000; Nomura et al., 2003; Bem and Rinzel, 2004).

Models also suggest that electrical synapses can promote anti-synchrony by preferentially passing the slower afterhyperpolarization that follows a spike. This causes electrical synapses to function as reciprocal inhibitory synapses, which optimizes firing when the neurons are 180° out of phase (Sherman and Rinzel, 1992; Bem and Rinzel, 2004; Ostojic et al., 2009; Vervaeke et al., 2010). Similarly, modeling by Ostojic et al. (2009) suggests that electrical synapses facilitate in-phase synchrony when they are primarily excitatory but facilitate bistable synchrony and asynchrony when they are primarily inhibitory; these models also suggest that electrical synapses are best able to promote synchrony when neurons are active at their resonant frequency. In addition, models by Pfeuty et al. (2003) demonstrate that electrical synapses promote anti-synchrony when there is a strong persistent sodium current and weak potassium current, but promote synchrony when there is a strong persistent potassium current. Other models suggest that generation of either synchronous or anti-synchronous activity require reciprocal GABAergic connections along with electrical synapses (Pfeuty et al., 2003; Bem et al., 2005; Gibson et al., 2005; Lau et al., 2010).

Although anti-synchronous firing between coupled neurons is a robust phenomenon across a wide spectrum of parameter space, experimental evidence for this phenomenon is notably lacking. One example of simulated electrical synapses and reciprocal GABAergic synapses, implemented using dynamic clamp in snail neurons, was able to produce anti-synchrony. Further, stimulation of sparse mossy fiber inputs to Golgi cells transiently switches coupled neurons from spiking synchronously to spiking asynchronously *in vitro* (Vervaeke et al., 2010; Figure 1B). However, anti-synchronous firing in coupled Golgi neurons was not observed *in vivo* (van Welie et al., 2016).

Contrary to the persistent thread of evidence connecting electrical synapses to synchronization, a few studies have suggested that electrical synapses fail to play a central role in synchronizing spiking in certain systems. In cortical inhibitory neurons, knockout of connexin36 did not impair synchrony of gamma activity, and synchrony of gamma activity was not correlated with electrical synapse strength (Salkoff et al., 2015; Neske and Connors, 2016).

### Beyond Synchrony

#### Excitation

As mentioned above, by passing depolarizations between coupled neurons gap junctions can act as excitatory synapses (Figure 1E). Several studies have identified electrical synapses as actors in lateral excitation, resulting in enhanced sensitivity to stimuli or neural input. Retinal ganglion cells exhibit increased responses to low-contrast and moving stimuli due to lateral excitation from electrical synapses between bipolar, amacrine, or ganglion cells (Trenholm et al., 2013; Kuo et al., 2016). Moreover, excitatory electrical relay from AII amacrine cells to rod bipolar cells is critical for rod-mediated responses in ganglion cells (Güldenagel et al., 2001; Deans et al., 2002). For guinea pigs, the receptive fields of ON center medium retinal ganglion cells (RGCs) are effectively expanded by lateral excitation from electrically coupled ON center α-RGCs (Puller et al., 2020). Under scotopic conditions, electrically coupled directionally sensitive ganglion cells (DSCGs) broaden their tuning for their preferred direction, while uncoupled DSCGs tuning remain stable. Consequently, coupled DSCGs enhance their detection of movement at the cost of discrimination of movement direction through lateral excitation (Yao et al., 2018). It is suspected that coupled DSCGs could potentiate coupling under scotopic conditions to induce that effect, but it remains to be directly measured. In the olfactory bulb, mitral cells enhance reactions to odorants through electrical lateral excitation (Christie and Westbrook, 2006). In addition, depolarizing a Golgi cell in cerebellum increases firing rate in its neighbors. Glutamate uncaging experiments in conjunction with gap junction blockers revealed that gap junctions helped compensate for the decay of the many chemical synaptic inputs at distal dendrites (Vervaeke et al., 2012).

Direct excitatory drive by electrical synapses is a physiological certainty in several cases. Strong electrical synapses between TRN neurons enable bursts in one cell to drive a spike in its coupled neighbor (Parker et al., 2009; Haas et al., 2011). Similarly, Curti et al. (2012) demonstrated the excitatory power of strong electrical synapses in the mesencephalic trigeminal nucleus. In a pair with a coupling coefficient of 0.51, spiking in the presynaptic cell was sufficient to drive spiking in the postsynaptic cell. Interneurons in the stratum lacunosum moleculare of the hippocampus can generate large depolarizations, up to 10 mV, in their coupled neighbors when they burst, due to slower depolarizations passing the gap junction (Zsiros et al., 2007). Goldfish club endings excite Mauthner cells through a mixed chemical and electrical synapse to trigger an escape reflex. The electrical synapses at the club ending experience a diverse array of plasticity mechanism, which may finely tune how the Mauthner cell responds to different auditory inputs (Pereda et al., 1992, 1994, 1998, 2004). M5 Intrinsically photosensitive retinal ganglion cells (ipRGCs) directly stimulate GABAergic amacrine cells through gap junctions (Pottackal et al., 2021). The amacrine cells then inhibit M5 and M4 ipRGCs; in this circuit, electrical synapses function as the excitatory component of feedback and feedforward inhibition. Striatal medium spiny neurons depolarize cholinergic interneurons through gap junctions, and electrical synapses contribute to the tonic activity observed in cholinergic interneurons (Ren et al., 2021). Electrical synapses also function to excite cortical parvalbumin neurons and cooperate with NMDAR inputs to drive bursts (Lee et al., 2021). In the TRN, spiking depolarizes coupled neighbors, and potentiation of an electrical synapse can transform a synapse from previously subthreshold to spike-driving (Fricker et al., 2021). Modeling of thalamocortical inhibitory feedback circuits revealed that excitation through electrical synapses in the TRN can affect the temporal separation of inputs relayed to cortex by thalamus (Pham and Haas, 2018). In this scenario, one TRN cell receiving earlier input from thalamus drives a coupled TRN cell to spike, which then delays spiking in its other thalamic cells. The degree of separation is directly tied to the strength of the electrical synapse, which can increase separation between spikes in thalamic relay cells by tens of ms. In a feed forward model circuit, powerful electrical synapses can drive coupled interneurons to spike and shorten the integration window for downstream cortical neurons (Pham and Haas, 2019).

Further, electrical synapses are capable of passing subthreshold chemical synaptic events. Subthreshold EPSPs in dorsal cochlear nucleus fusiform cells excite coupled stellate cells, and that excitation can be sufficient to drive spikes in the stellate cells (Apostolides and Trussell, 2014). Interneurons in the stratum lacunosum moleculare are excited by GABA due to HCO_3_^–^ leaving the cell (Perkins and Wong, 1996); excitatory GABAergic postsynaptic potential are passed through the gap junction. Inhibitory Renshaw cells in the spinal cord also excite each other by passing cholinergic EPSPs through gap junctions (d’Incamps et al., 2012). Thus, subthreshold chemical synaptic inputs in one cell are computational relevant in its electrical coupled neighbor and may be an understudied feature of electrical synapses.

#### Inhibition

By passing hyperpolarizations such as AHPs or IPSPs between coupled neurons, gap junctions act as inhibitory synapses (Figure 1D). Due to the low-pass filtering that results from current flowing across two cell membranes, slower signals are preferentially transferred through electrical synapses compared to faster events such as action potentials. In the case of spikes, slow AHPs are passed more effectively than the spike itself, making the event as a whole net inhibitory to the coupled cell. This effect was noted in fast spiking cortical interneurons (Galarreta and Hestrin, 2001). Inhibition from electrical synapses can be compounded with an accompanying inhibitory chemical synapse between coupled neurons, enhancing the inhibitory effect after spiking (Galarreta and Hestrin, 2001). The spike waveform in the presynaptic cell is critical in determining whether electrical synapses will excite, inhibit, or both after a spike. Spikes with larger half widths or broad depolarizations underlying a burst are better at exciting the postsynaptic cells, while fast spikes and large AHPs are net inhibitory. Gibson et al. (2005) demonstrated that fast-spiking inhibitory neurons send effective inhibition after spiking, while low-threshold spiking neurons primarily depolarize the postsynaptic neurons after spiking. Membrane potential also affects the net valence of post synaptic potentials from electrical synapses *via* differences in postsynaptic membrane conductances. Fast-spiking neurons almost exclusively depolarize postsynaptic cells after spiking when the postsynaptic neuron is hyperpolarized, but they primarily inhibit depolarized postsynaptic cells (Otsuka and Kawaguchi, 2013).

Inhibition carried by gap junction-mediated AHPs was also described in Golgi interneurons (Vervaeke et al., 2010; Yaeger and Trussell, 2016; Hoehne et al., 2020). Interestingly, in this case electrical synapse inhibition was shown to function similar to feedforward inhibition. Stimulation of excitatory parallel fiber inputs to electrically coupled basket cells results in an EPSP followed by electrically mediated inhibitory postsynaptic potential. The inhibition delivered by electrical synapses in this case sharpens the EPSPs that precede them and reduces temporal summation, hallmarks of feedforward inhibition (Hoehne et al., 2020). In striatum, burst firing between 25 to 60 Hz of fast-spiking interneurons inhibits spiking in coupled neighbors by transferring AHPs (Russo et al., 2013). In the dorsal cochlear nucleus, fusiform cells are able to transfer subthreshold synaptic voltage fluctuations to stellate cells *via* electrical synapses (Apostolides and Trussell, 2014). EPSPs in fusiform cells drive depolarization followed by a hyperpolarization caused by closing HCN channels (Apostolides and Trussell, 2014). Both the depolarizing and hyperpolarizing aspects of the chemical postsynaptic potential in the fusiform cell is transferred to stellate cells through the gap junction. The ability for electrical synapses to hyperpolarize neurons and inhibit spiking is an important aspect of their function that should not be overlooked.

#### Shunting

Unlike chemical synapses, electrical synapses passively contribute to the excitability of neurons without requiring activation of the synapse for that effect. When one neuron in an electrically coupled pair is stimulated, the gap junction to the neighboring cell shunts current away and renders the first neurons less likely to spike (Llinas et al., 1974; Van Der Giessen et al., 2008; Hjorth et al., 2009; Chatzigeorgiou and Schafer, 2011; Kawano et al., 2011; Rabinowitch et al., 2013; Figure 1C). Consequently, each hyperpolarized neuron in a coupled network inhibits spike generation, and depolarization of those neurons lifts that opposition. Shunting by electrical synapses can be especially impactful when one neuron is coupled to many other neurons. In such a network, a central neuron is strongly inhibited from spiking until a threshold of neighbors are also depolarized (Chatzigeorgiou and Schafer, 2011; Rabinowitch et al., 2013), effectively offering a mass-coincidence detection or insurance against erroneous responses.

Moreover, regulation of electrical synapse strength affects the overall excitability of a network. Coupled nNOS-1 amacrine cells in retina release nitric oxide in response to light, which consequently decouple the amacrine cells. Decoupling the amacrine cells increases their individual excitability by reducing the shunting of current to its neighbors (Jacoby et al., 2018).

#### Coincidence Detection

The same principles that enable electrical synapses to facilitate synchrony also allow them to act as coincident detectors. Typically, coincidence detection is conceptualized as the summation of near-simultaneous excitatory chemical inputs to one cell, driving spiking only when they are sufficiently coincident. Inputs arriving through electrical synapses can work in the same additive fashion. In addition, as mentioned earlier and in contrast to excitatory synapses, inactive electrically coupled neurons shunt current away from active neurons until they become depolarized themselves. As a result, electrical synapses can act as detectors of coincident depolarization between coupled neurons by effectively checking if both neurons in a pair are simultaneously depolarized before allowing spike generation. Furthermore, the spikelet generated by one neuron can drive spiking in a peri threshold neighbor, ensuring that coincident inputs are likely to result in both neurons spiking.

Galarreta and Hestrin (2001) injected current simulating subthreshold EPSPs into coupled cortical fast spiking interneurons, which drove spikes when they occurred within 1 ms, but failed to drive spikes when EPSPs were separated by 5 ms. Subthreshold current injections to each neuron of a pair that are mismatched in time fail to produce spiking in coupled amacrine cells, but generate spikes in both coupled cells when applied to both cells in a pair simultaneously (Veruki and Hartveit, 2002b). Similarly, Trenholm et al. (2013) stimulated one retinal ganglion cell with light and a coupled neighbor with current injection. Weaker light stimuli failed to drive spikes unless there was coincident stimulation of the coupled neighbor (Trenholm et al., 2013). In Golgi basket cells, simultaneous stimulation of both cells in a coupled pair has greater action potential probability compared to stimulation of one cell alone (Alcami, 2018). Computational modeling of striatal inhibitory neurons showed that electrical synapses reduce the overall firing of neurons in the circuit, but that effect was minimized when coupled neurons received coincident inputs (Hjorth et al., 2009). Experiments in *C. elegans* have found that RIH neurons act as a hub with electrical synaptic connections to multiple neural pathways; coincident stimulation from electrically connected partners were necessary to sufficiently stimulate RIH neurons and facilitate *C. elegans* escape response after a nose touch (Chatzigeorgiou and Schafer, 2011). Future modeling of the nose-touch-response circuit supports that electrical synapses from multiple pathways shunt current and make RIH neurons less responsive, while multiple simultaneous active neurons promote activation of RIH neurons and consequently the escape response (Rabinowitch et al., 2013). Taken together, the combined ability of electrical synapses to shunt current when a neuron is inactive, and promote activity when it is active, helps to cause synchronous activity and acts as detector of coincident activity across multiple neurons.

#### Signal to Noise Ratio

Neurons are subject to a variety of stochastic factors, including the random opening and closing of ionic channels and spontaneous synaptic activity. A fundamental task for the nervous system is to distinguish between signal and noise. Electrical synapses that shunt current and decrease excitability implement the functional effect of noise dampening (Figure 1F). Computational modeling supports the possibility of this mechanism for noise reduction *via* electrical synapses (Lamb and Simon, 1976; Vardi and Smith, 1996; Usher et al., 1999; DeVries et al., 2002; Medvedev, 2009).

Improvement of signal to noise ratio by electrical synapses has been investigated in multiple systems. Noise was measured in retinal preparations by recording membrane fluctuations of AII amacrine cells in complete darkness; noise was greater in amacrine cells with genetically knocked out connexin36 (Dunn et al., 2006). In the fish olfactory bulb, the variance of mitral cell response to an odorant increases with pharmacological blockade of gap junctions (Zhu et al., 2013). Electrical synapses suppress membrane oscillations entirely in drosophila lobula plate tangential cells, likely *via* shunting noisy intrinsic currents of in the cell (Ammer et al., 2022). It has also been inferred that electrical synapses improve signal to noise ratio in the fly olfactory bulb (Kazama and Wilson, 2009; Yaksi and Wilson, 2010) and monkey locus coeruleus (Usher et al., 1999). However, experiments comparing noise in the presence and absence of electrical synapses in those systems are still needed.

#### Asymmetry

Bidirectional flow of current is a notable distinguishing property of electrical synapses. Yet the magnitudes of signals relayed in each direction quite often show some degree of direction dependence. In the mammalian nervous system, asymmetry of synapses has been noted in neocortex (Galarreta and Hestrin, 2002), TRN (Haas et al., 2011; Sevetson and Haas, 2015; Zolnik and Connors, 2016), inferior olive (Devor and Yarom, 2002), cerebellum (Mann-Metzer and Yarom, 1999; Alcami and Marty, 2013; Szoboszlay et al., 2016), dorsal cochlear nucleus (Apostolides and Trussell, 2013), and mesencephalic trigeminal nucleus (Curti et al., 2012). Asymmetry could result from heterotypy of gap junction channels and plaques, where oligomerization or docking of different connexin or innexin proteins can asymmetrically pass current (Bukauskas et al., 1995; Phelan et al., 2008; Rash et al., 2013), and differences in hemichannel scaffolding (Marsh et al., 2017) could also contribute to the rectifying ability of electrical synapses. Even for homotypic gap junctions based on connexin36, which are largely voltage-independent (Srinivas et al., 1999), differences between cell properties, such as input resistance or cable properties, create functional asymmetry for signals sent across the gap junction (Mann-Metzer and Yarom, 1999; Veruki and Hartveit, 2002a; Nadim and Golowasch, 2006; Alcami and Marty, 2013; Amsalem et al., 2016; Mendoza and Haas, 2022). Furthermore, those different sources of asymmetry can also compensate for or exacerbate genuine junctional asymmetry between electrical synapses (Mendoza and Haas, 2022). For example, at the Mauthner mixed synapse, heterotypic asymmetrical gap junctions composed of connexin34.7 and connexin35 preferentially pass current from the Mauthner cell to the club endings. At that synapse, rectification compensates for the large dendrite of the Mauthner cell, making the electrical synapse more functionally bidirectional (Rash et al., 2013).

Asymmetry of electrical synapses directly impacts their function. Full rectification can create nearly unidirectional communication, such as in the giant motor neuron of crayfish or pyloric circuit of the spiny lobster (Furshpan and Potter, 1957, 1959; Graubard and Hartline, 1987; Johnson et al., 1993). Voltage-dependent gating of gap junction channels allows crayfish presynaptic giant interneurons to unidirectionally excite the giant motor neuron and engage an escape reflex (Jaslove and Brink, 1986). For the pyloric circuit of the spiny lobster, the rectified synapse allows the lateral pyloric neuron to drive the pyloric neuron to burst, and then send a delayed chemical inhibitory signal to terminate the burst (Graubard and Hartline, 1987; Mamiya et al., 2003). In the dorsal cochlear nucleus (Apostolides and Trussell, 2014), near-complete asymmetry is a result of input resistance mismatch, and results in fusiform cells driving spikes in more-compact stellate cells. Asymmetry in this case ensures recruitment of local inhibition within the circuit even during subthreshold excitation of the fusiform cells.

For more moderate asymmetry, modeling results reveal an effect of asymmetry on detailed spike timing, even to the extent of reversing spike order in a coupled cells (Sevetson and Haas, 2015), and controls the phase of synchronous rhythmic activity (Mendoza and Haas, 2022). Modeling of larger networks shows that rectifying electrical synapses can improve the robustness of rhythmic activity (Gutierrez et al., 2013). Because electrical synapse plasticity has been shown to systematically alter the degree of asymmetry (Haas et al., 2011; Fricker et al., 2021), asymmetry may be actively regulated in order to tune spike timing and network activity. Collectively, our understanding of the function of asymmetry is still limited, but its potential impacts are worthy of additional investigation.

### Future of Electrical Synapse Study

The study of electrical synapse function has a dense focus on the contribution of electrical synapses to synchronization of activity between coupled pairs. This attention is merited, and there is ample evidence to support this function, especially when neurons in a coupled network receive prolonged, simultaneous excitation. The next decade of research would greatly benefit from studies focusing on how electrical synapses in different systems affect transient inputs, and from studies that look at how temporal variation in the activity of multiple electrically coupled neurons in a network affect activity. In *C. elegans*, a hub and spoke model has been proposed, where a central neuron integrates the information from multiple electrical synapses to determine whether to fire. Such a computational process has not yet been explored in mammalian electrical networks.

One limitation to understanding the functions of electrical synapses within and beyond a network is that the gold standard for identification and measurement of coupling between neurons is dual whole-cell recordings, which is difficult and limited to two cells at a time. In this manner, the network is neglected. Extracellular methods, such as multi-electrode arrays, field recordings, and wide-scale imaging of fluorophores overcome this limitation, but do not reveal which neurons are coupled in a network or the strengths or spatial distributions of their coupling. One could hope that cell-specific optogenetics could mitigate the barrier (Dakin and Li, 2006; Qiao and Sanes, 2016), and some all-imaging approaches have identified GJ-mediated signals (Tian et al., 2021). In computational studies of neural networks, electrical synapses are most often neglected entirely, which we regard as a vast missed opportunity.

Improved imaging could bring new insight into the function of electrical synapses. Currently, we lack a dye that can pass through connexin36-based gap junctions and be imaged in live tissue, which limits our ability to identify and image specific gap junctions. A new GCaMP connexin36 hybrid gene has been created and used in HeLa cells (Moore et al., 2020). It is possible that replacement of the connexin36 gene with the GCaMP connexin36 hybrid would allow researchers to look at localized activity in the dendrites and near the gap junction. However, this is early speculation, and the viability of such a method needs to be vetted. Another major complication is differentiating connexin36 in the cytoplasm from connexin36 that forms gap junction pores.

A recent innovation has created novel connexin genes through an iterative mutation approach. Mutated perch connexin34.7 and connexin35 proteins form exclusively heterotypic gap junctions (Ransey et al., 2021a) resulting in asymmetrical electrical synapses (Ransey et al., 2021b), and expression of each protein can be directed toward specific cell types. This technique could be useful for interrogating how the addition or substitution of asymmetric electrical synapses modifies a circuit and behavior.

Electrical synapses have profound and complicated impacts on the processing of neural signals. The functional consequence of electrical synapses is collectively impacted by the membrane voltages of pre- and post-synaptic neurons, the waveform of spikes, the frequency of spiking, and their location on dendrites. Wherever electrical synapses are present, their function should be thoroughly interrogated. Moreover, it is important for connectome projects (Van Essen et al., 2013; Oh et al., 2014; Zhang et al., 2019) to include electrical connections in addition to chemical connections to achieve a whole picture of neural communication pathways.

## Author Contributions

JH and MV contributed equally to conceptualization and writing. Both authors contributed to the article and approved the submitted version.

## Conflict of Interest

The authors declare that the research was conducted in the absence of any commercial or financial relationships that could be construed as a potential conflict of interest.

## Publisher’s Note

All claims expressed in this article are solely those of the authors and do not necessarily represent those of their affiliated organizations, or those of the publisher, the editors and the reviewers. Any product that may be evaluated in this article, or claim that may be made by its manufacturer, is not guaranteed or endorsed by the publisher.
